# Estimating the risk of aflatoxin-induced liver cancer in Tanzania based on biomarker data

**DOI:** 10.1371/journal.pone.0247281

**Published:** 2021-03-11

**Authors:** Martin E. Kimanya, Michael N. Routledge, Emmanuel Mpolya, Chibundu N. Ezekiel, Candida P. Shirima, Yun Yun Gong

**Affiliations:** 1 School of Life Sciences and Bioengineering, Nelson Mandela African Institution of Science and Technology, Arusha, Arusha Region, United Republic of Tanzania; 2 School of Medicine, University of Leeds, Leeds, West Yorkshire, United Kingdom; 3 School of Food and Biological Engineering, Jiangsu University, Zhenjiang, Jiangsu Province, China; 4 Department of Microbiology, Babcock University, Ilishan Remo, Ogun State, Nigeria; 5 Tanzania Bureau of Standards, Dar es Salaam, Dar es Salaam Region, United Republic of Tanzania; 6 School of Food Science and Nutrition, University of Leeds, Leeds, West Yorkshire, United Kingdom; Centre de Recherche en Cancerologie de Lyon, FRANCE

## Abstract

Evidence about the magnitude of the aflatoxin menace can help policy makers appreciate the importance of the problem and strengthen policies to support aflatoxin mitigation measures. In this study, we estimated aflatoxin-induced liver cancer risk in 2016 for Tanzania and used the information to estimate the health burden due to the aflatoxin exposure in the country. The burden of aflatoxin-induced liver cancer was assessed based on available aflatoxin biomarker data from a previous epidemiology study, hepatitis B virus infection prevalence and population size of Tanzania in 2016. The health burden due to aflatoxin-induced liver cancer was estimated using disability adjusted life years (DALYs). The aflatoxin exposures ranged from 15.0–10,926.0 ng/kg bw/day (median, 105.5 ng/kg bw/day). We estimated that in 2016 there were about 1,480 (2.95 per 100,000 persons) new cases of aflatoxin-induced liver cancer in Tanzania and assumed all of them would die within a year. These morbidity and mortality rates led to a total loss of about 56,247.63 DALYs. These results show, quantitatively, the cases of liver cancer and related deaths that could be avoided, and the healthy life years that could be saved, annually, by strengthening measures to control aflatoxin contamination in Tanzania.

## Introduction

Aflatoxins are fungal metabolites, primarily produced by *Aspergillus*, mainly *A*. *flavus* and *A*. *parasiticus*, in foods such as maize, groundnuts, tree nuts and spices, and which have impacted human health for many years. There are four types of aflatoxin that are important in health and agriculture: aflatoxin B_1_ (AFB_1_), B_2_ (AFB_2_), G_1_ (AFG_1_) and G_2_ (AFG_2_). AFB_1_ is the most common and most toxic of the four types. Intake of high doses of aflatoxins can cause acute aflatoxicosis whereas intake of low to moderate doses over a long period can result in immune suppression, impaired growth, low birth weight and liver cancer [1–3]. The largest and most well-known of these effects is liver cancer. The International Agency for Research on Cancer (IARC) has rated aflatoxins as Group 1 liver carcinogen, meaning that there is sufficient evidence of carcinogenicity in humans [4]. It is estimated that in 2018, liver cancer was the 8^th^ most common-incident cancer and the 4^th^ most common cause of cancer deaths, worldwide [3]. According to the report, there were 841,080 incident cases of liver cancer and 781,631 deaths, globally. Hepatocellular carcinoma (HCC) accounts for 75–85% of the global liver cancer cases [3]. The main risk factors for HCC (chronic infection with hepatitis B virus -HBV or hepatitis C virus -HCV, aflatoxin exposure, or other lifestyle factors including alcohol intake, smoking, obesity, and type 2 diabetes) vary by region or country but in East Africa region (Tanzania inclusive) the main risk factors are HBV infection and aflatoxin exposure [3]. The role of aflatoxin exposure in liver cancer development is well documented [5,6]. The risk of developing liver cancer in individuals exposed to aflatoxins is 30-fold higher in individuals who are hepatitis B virus positive (HBV+) compared to those who are HBV negative (HBV-) [5,6]. Unfortunately, both HBV infection and aflatoxin exposure are common in poor nations including those in Africa [3,5,7,8].

It is estimated that 40% (59,900 of the 155,000) global annual cases of aflatoxin-induced liver cancer occur in Africa [7]. To arrive at this conclusion, the risk assessors estimated the global burden of aflatoxin induced-liver cancer, using food aflatoxin levels, consumption of aflatoxin contaminated foods and HBV prevalence in several countries, globally. The assessors found that up to 28.2% of the annual liver cancer cases, globally, are linked to aflatoxin exposure. Countries within sub-Saharan Africa are among the most important nations for aflatoxin-related liver cancer because the climatic conditions and poor food growing and storage practices in Africa favor growth and proliferation of the aflatoxin-producing *Aspergillus* species [8,9]. The *Aspergillus* species can therefore colonize and produce aflatoxins in a wide variety of food commodities, including maize, rice and groundnuts, which are staple foods in Africa [9]. In the context of Tanzania, studies implicate maize as the main source of aflatoxin exposure. Smith and Subandoro [10] reported that for the whole of Tanzania, the staple food most highly consumed is maize, of which nearly 400 grams a day per person is consumed and that the second most important staple is cassava, followed by rice. In a study by Makori et al. aflatoxins were detected in 42.5% of home-made maize based complementary flours [11]. The levels ranged from 0.40–2,129 μg/kg and in 30.6% of samples, were above the national regulatory limit of 10 μg/kg. Another study, Kamala et al., reported 50% of all maize based food samples intended for human consumption were contaminated with aflatoxins and in the contaminated samples, 28% exceeded the limits of 5μg/kg [12].

Kimanya et al. [13] detected aflatoxins in 18% of maize samples from four regions of Tabora, Ruvuma, Kilimanjaro, and Iringa in Tanzania, at levels of up to 158 μg/kg. Twelve percent of the samples exceeded the Tanzania regulatory limit (10 μg/kg) for total aflatoxins. A risk assessment performed by Shirima et al. [14] in three of these regions (Iringa, Tabora and Kilimanjaro), found high prevalence of chronic aflatoxin exposures (measured as aflatoxin albumin -AF-alb adducts in blood) in young children. The exposure levels were associated with maize diet, increased with age, and varied with season and location [14]. The increase in exposure with age reflects the combined effect of increased consumption of contaminated family food as children grow whereas the variation in exposure reflects the seasonal and regional variation of aflatoxin contamination in food. The seasonal variation would be expected because concentrations of aflatoxin increase during storage, and the regional variation because climatic conditions in Tabora are more favorable for aflatoxin formation in food compared to Iringa and Kilimanjaro [13].

Although measures to prevent and reduce aflatoxin contamination and exposure in food exist, there is low adoption of such measures in Africa. The low adoption of aflatoxin mitigation measures is partly attributed to limited appreciation, among policy makers, of the health and economic impacts of aflatoxins. Policy makers find it difficult to understand the extent to which aflatoxins affect the human society, especially in the case when death is not a significant outcome under chronic exposure [15,16]. Generating more evidence about the magnitude of the aflatoxin menace (such as incidences of liver cancer and deaths associated with the disease) can help policy makers appreciate the importance of the problem and strengthen policies to support aflatoxin mitigation measures.

Cancer registration system in Africa is, despite development, still of limited capacity. Cancer incidence data, either crude or organ specific, is not fully available nor accurate in these countries. It is thus not possible to estimate aflatoxin-induced liver cancer based on registration figures on cancer incidence in a specific population such as Tanzania.

The risk of aflatoxin-induced liver cancer in a nation can be estimated on the basis of the two risk factors of HBV infection and aflatoxin exposure [5,6]. HBV prevalence rates for various nations as published from the year 2000 were summarized in [7,17]. Aflatoxin exposure in an individual can be estimated by combining the daily per capita consumption of food with aflatoxin content in the consumed food and dividing the result by body weight of the individual. However, estimation of dietary aflatoxin exposure is cumbersome and gives unreliable results due to many factors including difficulties in estimation of food consumption and inaccuracy in measuring aflatoxin contamination in the consumed food. The use of biomarkers of aflatoxin exposure is a more reliable means of estimating aflatoxin exposure as it directly measures internal doses in individuals [18–20]. We here utilize a dataset of aflatoxin biomarkers for Tanzania collected by Shirima et al [14] to assess the burden of aflatoxin-induced liver cancer in the country. The outcomes of this assessment may be used to advocate for formulation of appropriate policies for control of aflatoxins in Tanzania and other countries of Africa with similar aflatoxin exposure patterns.

## Materials and methods

### Source of the child dataset

The AF-alb adduct biomarker dataset employed in this study was extracted from an epidemiological study conducted in three villages of Kigwa, Nyabula and Kikelelwa located in agroecologically different and distant zones of Tanzania [14]. Kigwa is located in Tabora region (western zone), Nyabula in Iringa region (southern zone) and Kikelelwa in Kilimanjaro region (northern zone) of Tanzania [13]. The three sites in the study are considered as representative of aflatoxin exposure in Tanzania.

### Regional and sampling point distribution of the children

The AF-alb adduct levels were determined in blood samples collected from the group of children at three time points; at recruitment (during maize harvest season when newly harvested maize was consumed) and six and 12 months after recruitment (during the season when stored maize was consumed) to account for variation in exposure due to both age and season. The number of subjects from whom the AF-alb measures were obtained as well as the number of samples collected at each sampling point are described in Shirima et al [14]. Briefly, 41, 57 and 49 children were studied for aflatoxin biomarker from Tabora, Iringa and Kilimanjaro, respectively. The exposure biomarker levels of these children were studied at the three time points as described above, with a small number of loss at follow-up. In total, 436 AF-alb measures were generated from blood samples collected at the three time points. The distribution of the AF-alb measures according to region and sampling points is shown in Table 1.

**Table 1:** Regional and sampling point distributions of the AF-alb measures.

Sampling point	Tabora	Iringa	Kilimanjaro	Overall
**Recruitment (n)**	41	57	49	147
**6 months after recruitment (n)**	37	55	54	146
**12 months after recruitment(n)**	36	54	53	143
**Overall (n)**	114	166	156	436

### Characteristics of the children

Characteristics of the children were described in Shirima et al. [14]. Briefly, at recruitment, all the children were already introduced to complementary foods. The foods were maize-based and given in form of a porridge. Other foods given to the children were groundnuts, banana, potatoes, rice, finger millet, beans, cassava, meat, fresh cow’s milk, eggs, vegetables, and fruits. Levels of the biomarker of exposure increased with age. At recruitment, AF-alb was detected in 67% of the children (6–14 months old) with a geometric mean concentration of 4.7 pg adducts/mg of albumin. The levels increased to 84% and 12.9 pg AF-alb adducts/mg of albumin 6 months after recruitment, and to 99% and 23.5 pg AF-alb adducts/mg of albumin at 12 months after recruitment, respectively. About 96% of the children were from subsistence farming households. Eighty nine percent mothers to these children had completed primary education, and 78% were married. Families in the Kilimanjaro region were socioeconomically wealthier than those in Tabora or Iringa.

#### Analysis method

The AF-alb adducts were analysed using an ELISA-based method at the University of Leeds (UK). The ELISA method has been previously described [20]. The limit of detection (LOD) for the assay was 3 pg AF-alb adducts/mg of albumin. Values below the LOD were assigned half of the LOD at 1.5 pg AF-alb adducts/mg of albumin.

Summaries of the distribution of the AF-alb dataset are shown in Table 2.

**Table 2:** Regional and size distributions of the aflatoxin biomarker data.

	Tabora (n = 114)	Iringa (n = 166)	Kilimanjaro (n = 156)	Overall (n = 436)
**% > LOD**	97	79	78	84
**Range (pg/mg albumin)**	3.00–1092.60	3.00–253.70	3.00–631.20	3.00–1092.60
**Median (pg/mg albumin)**	24.25	6.10	10.55	10.55

LOD = 3 pg AF-alb adducts/mg of albumin.

#### Conversion of units

The overall dataset (Table 2) was used for assessment of national level exposure and health impact of aflatoxins. To give insight of the exposures and health impacts in the regions (Tabora, Kilimanjaro and Iringa) from which the biomarker data were obtained, assessment was also carried out for each of the regions. All the biomarker measures were converted into exposures in μg/kg bw/day (for an individual of 70 kg) by using the method described by Shephard [21]. Specifically, a biomarker measure (in pg AF-alb/mg albumin) was divided by 100 in order to obtain an equivalent exposure in aflatoxin B_1_ (AFB_1_) μg/kg bw/day. Shephard estimated that 100 pg AF-alb adducts/mg of albumin represents exposure of 1,000 ng of AFB_1_/kg bw/day [21]. The author estimated this relationship by finding a mean of 107 pg AF-alb adducts/mg of albumin per 1 μg AFB_1_/kg bw/day estimated by Gan et al. [22] and 94 pg AF-alb adducts/mg of albumin per 1 μg AFB_1_/kg bw/day recalculated from a previous estimate by Wild et al. [23].

### Estimation of population risk for aflatoxin-induced liver cancer

The population risk (cases per 100,000 people) for aflatoxin-induced HCC was estimated using the approach described by the Joint FAO/WHO Expert Committee on Food Additives (JECFA) [5,6]. The JECFA provided specification of HCC potency factors for aflatoxins as 0.01 cases per 100,000 persons per year per ng of aflatoxin/kg bw/day for individuals without chronic HBV infection and 0.30 corresponding cases for individuals with chronic HBV infection. The calculation employed Eq 1.

Populationrisk(casesper100,000persons)=Medianexposurevalue*AverageHCCpotency,

Where;AverageHCCpotency=(HBV+prevalence*HCCpotencywithHBV+)+(HBV−*HCCPotencywithHBV−) (1)

This assessment employed the median exposures described in Table 2 (after exposures had been converted into ng/kg/bw/day) and the HBV prevalence of 4.2% for Kilimanjaro, 4.3% for Iringa and 6.2% for the nationwide population [24]. Since there are no published HBV+ prevalence data for Tabora, the nationwide prevalence of 6.2% was used for this region.

### Estimation of annual incidence of aflatoxin-induced liver cancer

The annual incidence of aflatoxin-induced liver cancer for Tanzania was estimated based on the median population risk as estimated above, and the population size for Tanzania in 2016 which was 50,144,175 [25]. Likewise, annual incidences of aflatoxin-induced liver cancer for Iringa, Kilimanjaro, and Tabora were estimated based on the calculated median population risks and respective population sizes of 984,882, 1,759,048 and 2,576,053 in 2016 [25].

### Estimation of the Disability Adjusted Life Years (DALYs)

Mean Disability Adjusted Life Years (DALYs) due to aflatoxin-induced liver cancer in Tanzania were calculated by multiplying the median annual incidence of aflatoxin-induced liver cancer, as estimated in this assessment, by DALYs per one case of all-cause aflatoxin liver cancer cases, calculated by this study. DALY is the summary measure used to give an indication of overall burden of disease. The sum of years of life lost and years lived with disability represents DALYs. One DALY can be interpreted as one lost year of “healthy” life.

The calculation of DALYs per capita (case) of all-cause liver cancer cases, was based on updated results from the Global Burden of Diseases (GBD) Project 2016 [26] for Tanzania. The GBD project provides summaries of DALYs, Years of Life Lost (YLL) and Years Lived with Disability (YLD) incidences of diseases for various locations on the globe. The reported number of all-causes liver cancer cases for Tanzania in 2016 is 1,623 and DALYs, 61,756. The case-fatality rate for liver cancer in Tanzania can be as high as 95% [3] making the number of deaths (mortality) almost equal to the total number of cases. Upon obtaining these numbers of all-cause liver cancer cases (1,623) and the total DALYs due to liver cancer (61,756), the number of DALYs per capita (case) of 38 was calculated by dividing the latter by the former.

## Results

### Aflatoxin exposures in ng per kg bw per day

The nationwide aflatoxin exposures ranged 15.0–10,926.0 ng/kg bw/day (median, 105.5 ng/kg bw/day). The highest median exposure was recorded for measures from Tabora (median, 242.5 ng/kg bw/day; range, 15.0–10,926.0 ng/kg-bw/day). Exposure ranges in Kilimanjaro were 15.0–6,312.0 ng/kg bw/day (median, 105.50 ng/kg bw/day) and in Iringa, 15.0–2,537.0 ng/kg bw/day (median, 61 ng/kg bw/day).

Eighty one percent (81%) of the exposures for Tabora exceeded the national median exposure of 105.5 ng/kg bw/day. Table 3 shows the regional variation in patterns of exposures per quartiles of the exposure distributions.

**Table 3:** Variation in patterns of exposures (ng/kg bw/day) according to regions.

Percentile	Tabora^x^	Kilimanjaro^y^	Iringa^z^	Tanzania^x^
**0.05**	42.0	15.0	15.0	15.0
**0.25**	105.0	30.0	31.0	37.0
**0.5**	242.5	105.5	60.5	105.5
**0.75**	447.0	252.0	121.0	270.5
**0.95**	2,386.0	1,506.0	492.0	1,591.5

^X^, = Average potency of 0.02798, ^y^, = Average potency of 0.02218; ^z^, = Average potency of 0.02247.

### The risk, cases and DALYs of aflatoxin induced liver cancer

The overall median (nationwide) population risk for aflatoxin-induced liver cancer was estimated to be 2.95 per 100,000 people. The median population risks (cases per 100,000 persons) varied from 1.37 (Iringa) to 6.79 (in Tabora). Based on these median population risks, the total number of aflatoxin-induced liver cancer cases per year as well as respective DALYs for Iringa, Kilimanjaro, Tabora and Tanzania, in general, were estimated and presented in Table 4.

**Table 4:** Estimated population risk, annual cases and DALYs for aflatoxin-induced liver cancer.

Location	Median exposure (ng/kg bw/day)	Population risk (cases per 100,000 persons)	Liver cancer cases in 2016	DALYs in 2016
**Iringa**	61.0	1.37	13	512.98
**Kilimanjaro**	105.5	2.34	41	1,564.14
**Tabora**	242.5	6.79	175	6,641.98
**Tanzania (nationwide)**	105.5	2.95	1,480	56,247.63

## Discussion

We estimated that in 2016, the population risk (cases per 100,000 people) for aflatoxin-induced liver cancer in Tanzania was 2.95. We also estimated that the population risk in the studied regions varied from 1.37 (in Iringa), through 2.34 (in Kilimanjaro) to 6.79 (in Tabora). The risks estimated for Iringa, Kilimanjaro, Tabora and Tanzania are similar to risks recently reported for Borgou (7.4) in Benin or Kano (1.4) in Nigeria or Bamako (2.0) in Mali [27]. All these countries have tropical climates which provide favorable conditions for mycotoxin contamination in foods (maize and groundnuts, in particular). The risk (6.79 cases per 100,00 people) estimated for Tabora is about two-fold of the nationwide risk (2.95 cases per 100,000 people). We have previously reported that contamination of aflatoxins in maize flour consumed by the children from whom the biomarker data were generated was much more common in Tabora compared to Iringa or Kilimanjaro [14]. Such differences between regions reflect differences in climate and soil conditions. Tabora is one of the semi-arid regions of Tanzania. Drought is one of the factors predisposing crops to fungal infection and mycotoxin formation. Further, most people in Tabora consume groundnuts as staple food, and the crops is susceptible to aflatoxin contamination [29].

We estimated that in 2016, Tanzania had 1,480 new cases of aflatoxin-induced liver cancer and that the total DALYs (healthy life years lost) due to these aflatoxin-induced liver cancer cases would be 56,247.63 (112 DALYs per 100,000 persons). These healthy life years could be saved if workable aflatoxin control measures are adopted in this country. From risk management point of view, strict food regulations and effective enforcement are the best strategies, where possible. But, in subsistence farming communities such as those in Tanzania, amongst many other mitigation methods explored, to date the best option to minimize the risk of aflatoxins contamination and exposure is adoption of good agricultural practices (including biocontrol technology) during pre-harvest and post-harvest stages of food management (including appropriate storage). Bandyopadhyay *et al*. reported that adoption of biocontrol has been proven to reduce aflatoxins by as much as 98% in staples (such as maize and groundnut) across several countries in Africa [9]. Also, it was demonstrated by Wu and Khlangwiset [16] for Nigeria that, at an upper estimate, if that technology is adopted up to 184,000 DALYs will be saved annually. Wu and Khlangwiset [16] showed further that if the post-harvest intervention package tried by Turner *at al*. [28] in Guinea is adopted in that country, 1,121 DALYs related to aflatoxin-induced liver cancer cases would be saved, annually. The post-harvest intervention package consisted of six components which are cheap and available to most Tanzanians. The components are education on hand-sorting of nuts, natural fibre mats for drying the nuts, education on proper sun-drying, natural-fibre bags for storage, wooden pallets on which to store bags, and insecticides applied on the floor of the storage facility under the wooden pallets [28]. These examples demonstrated the extent of healthy life years that can be saved in Nigeria and Guinea if those countries adopted these measures to prevent aflatoxin-induced liver cancer. Although the estimates for Nigeria and Guinea are not directly transferable to the Tanzania situation, they show that adoption of these cost-effective interventions requires fewer resources than can be served through minimized risks of aflatoxin exposure and liver cancer. As for setting and enforcing appropriate maximum limits for aflatoxins in food, Abt Associates Inc. estimated that the monetary loss due to aflatoxin-induced liver cancer in Tanzania can be reduced to $147 million if the country enforces its national limit of 5 μg/kg set for AFB_1_ in food [29]. However, it should be noted that implementation of standards is a challenge because majority of the people in developing countries like Tanzania consume their own grown foods or locally unpackaged foods, which are not formally regulated. Other options for minimizing the risk of aflatoxin contamination and exposure include diversification of diet to reduce consumption of aflatoxin prone foods, removal of toxins (through means like sorting and screening) at household level and vaccination of the general population against Hepatitis B and C Viruses to minimize the risk of liver cancer development [8].

We estimated the annual aflatoxin-induced liver cancer cases in Tanzania using biomarker data. In Tanzania, the aflatoxin biomarker data was derived from children. The use of these dataset was with a strong assumption that they represent the aflatoxin exposure in the entire populations of Tanzania in 2016. Nonetheless, it is worth noting that although Shirima *et al*. [14] noted that children may have higher intake of aflatoxin than adults, relative to their body size, a study in Uganda [30] found no significant difference in aflatoxin levels among children and adults. The use of data from children to estimate the burden of aflatoxin-induced liver cancer is also warranted by the fact that a recent study by the Africa Liver Cancer Consortium shows that liver cancer tends to develop at a younger age in Africa than in other regions of the world [31]. It is not possible to know the actual exposure that contributed to liver cancer incidence in 2016, but the biomarker data provides the best source of data for making an estimation. These data measure exposure over a period including different seasons and in three geographical regions, so give an integrated estimate of exposure in Tanzania.

The total number of aflatoxin-induced liver cancer cases per year was estimated to be 1,480. The burden of aflatoxin-induced liver cancer is consistent with the high rates of HBV infection and aflatoxin exposure in this country. The HBV+ prevalence applied for Tanzania is 6.2% [24] and aflatoxin exposure in this country is known to be high [14,32,33]. The subjects from whom the biomarker data were obtained consumed a diet that was predominantly maize based [14]. We previously reported a significant correlation between the dietary intake of aflatoxins and the blood aflatoxin biomarker levels [34]. It may also be seen from Table 3 that aflatoxin exposure varies among individuals in the country. The reasons for the variation were discussed in detail by Shirima *et al*. and were found to be largely due to local climate and dietary diversity [14]. Exposure is likely to be higher in areas where drought, high temperature, low soil fertility, pest infestation and other stresses are common, as these factors affect plant growth and vigor, thus increasing the likelihood of fungal infection, as well as the levels of aflatoxins produced by the *Aspergillus* fungi [9]. In addition, households that cannot afford a diversified diet are at a higher risk of aflatoxin exposure because their per capita consumption of aflatoxin prone foods is higher [10].

In addition to liver cancer, aflatoxin exposure is associated with other health effects such immune suppression [35] and stunting [14,36,37]. We estimated aflatoxin-related health impacts for liver cancer only, because this is the only endpoint for which a clear etiology mechanism is established [5,6]. We also estimated DALYs related to aflatoxin-induced liver cancer as an attempt to present the aflatoxin problem in a language that can be appreciated by policy makers. Therefore, the results presented here may be an underestimate of the total impact of aflatoxin contamination in Tanzania. The overall intention is to trigger more policy actions for mitigation of the aflatoxin problem in the country as well as other countries of Africa where aflatoxin exposure is a problem. This evidence is important because in the absence of aflatoxicosis outbreak it is very difficult for policy makers to understand the magnitude of the health impacts of chronic exposures to aflatoxins as the toxins are not visible and the impacts (such as liver cancer) manifest long after the exposure. We understand the importance of health-related economic impacts of aflatoxin exposure, but due to limited resources we could not include them in this assessment. The economic impacts include raised demand for medical services, falling labor efficiency, the time sufferers spend seeking medical attention, and the time spent by family members attending to the sick.

Liu and Wu estimated that up to 28.2% of all-cause liver cancer cases in a country can be attributable to aflatoxins [7]. Assuming the estimation applies to the Tanzania situation, the all-causes annual liver cancer cases for Tanzania would be as high as 5,248 (1,480*100/28.2). The Global Disease Burden Project of 2016 reported the total number of all-causes liver cancer for Tanzania as 1,623 [26]. The current analysis suggests aflatoxin exposure makes a substantial contribution to liver cancer in Tanzania. The country does not have a population-based cancer registry. The data available, on which cases in the Global Burden of Diseases project are based, was obtained from patients referred to the country’s national cancer hospital (The Ocean Road Cancer Institute), in Dar es Salaam. Thus, the total cancer cases are likely to be higher than reported in the Global Burden of Disease Project [38].

The assessment confirmed that aflatoxin exposures in Tabora and other regions where maize and groundnuts are consumed in larger quantities, are extremely high and the populations are at relatively higher risk of aflatoxin-induced liver cancer. The use of biomarkers of aflatoxin exposure made it possible for us to estimate the burden of aflatoxin-related liver cancer in Tanzania, using DALYs. The DALYs represent the healthy life years that could be averted, annually, by strengthening measures to control aflatoxin contamination in Tanzania. We recommend urgent financial investments for mitigation of the aflatoxin problems in Tanzania and other countries of Africa. The costs for mitigating the problem are expected to be by far lower than the benefits (health losses) estimated in this assessment.

## Supporting information

S1 Table (XLS)
